# Psychotic-like experiences and associated socio-demographic factors among pregnant women in each trimester in China

**DOI:** 10.3389/fpsyt.2022.927112

**Published:** 2022-09-23

**Authors:** Dali Lu, Shuangyan Qiu, Danxia Xian, Jingyu Zhang, Yan Zhang, Xiaocheng Liu, Weikang Yang, Xiaoqun Liu

**Affiliations:** ^1^Department of Pediatric Psychology, Shenzhen Longhua Maternity and Child Healthcare Hospital, Shenzhen, China; ^2^Shenzhen Longhua Maternity and Child Healthcare Hospital, Shenzhen, China; ^3^Department of Women and Children Health, Xiangya School of Public Health, Central South University, Changsha, China

**Keywords:** psychotic-like experiences, pregnant women, CAPE, EPDS, GAD-7, trimester

## Abstract

**Objective:**

Psychotic-like experiences (PLEs) are quite common in the general populations without a clinical diagnosis, but pregnant women have been neglected in earlier literature. This study aimed to investigate the prevalence and correlates of PLEs among pregnant women without previous psychiatric history in each trimester.

**Method:**

A total of 950 pregnant women participated in a cross-sectional survey, with social and demographic information collected. The Positive Subscale of Community Assessment of Psychic Experiences (CAPE) was used to measure PLEs, and the 7-item Generalized Anxiety Disorder Questionnaire (GAD-7) and the Edinburgh Postnatal Depression Scale (EPDS) were used to examine anxious and depressive symptoms, respectively. Logistic regression analyses were conducted to investigate the risk factors for pregnant women with PLEs.

**Results:**

In our study, 37.2% of the pregnant women in this sample experienced at least one episode of PLEs, while 4.3% reported “often” having PLEs. More pregnant women experienced PLEs, delusional experiences, and hallucinatory experiences in the first two trimesters than in the third trimester. Factors associated with a higher risk for more frequent PLEs include: rural setting, unplanned pregnancy, parity 1, and EPDS scores. High positive correlations were shown between frequency scores among experiences of PLEs and GAD-7 scores, EPDS scores.

**Conclusion:**

Episodes of PLEs are common in Chinese pregnant women; however, only a small proportion has persistent PLEs. It is vital to pay attention to women with psychosis risk in pregnancy.

## Introduction

Pregnancy is a major event in any woman's life. For a woman with, or who is susceptible to, severe mental illness, this transition might signal a time of unparalleled change. Previous studies have shown that non-psychotic mental disorders such as depressive disorders, anxiety disorders, and post-traumatic stress disorder are among the commonest morbidities of the perinatal period (1). This perinatal period including the pregnancy and post-partum period is also associated with an increased risk of severe mental disorders with psychosis, such as schizophrenia, affective psychosis, and bipolar disorder (2). Women with pre-existing mental disorders who become pregnant can be severely affected in the perinatal period, and women without pre-existing mental disorders can develop an onset during this period due to the alterations in the hormone levels, immunological factors, and onset of sleep deprivation among other factors (2, 3). Psychosis symptoms also can be severe in this perinatal period (1), and pose the risk to the safety of the mother and infant (4).

However, most studies on perinatal psychosis are about postpartum, and fewer studies are concerned about the pregnancy period. Psychosis during the pregnancy period can have adverse effects on the mother, her child, and her family (5, 6). Psychosis like experience (PLE) such as sporadic delusions or hallucinations, are subthreshold, non clinical form of psychosis, that occur commonly in the community and are mostly transient in nature (7). PLEs may predict the onset of psychosis and subsequent non-psychotic disorders (5, 8). Some women with PLEs during pregnancy and/or after delivery may develop postpartum psychosis (9). So far, the study of PLEs in perinatal women has been neglected, only three studies are concerned about PLEs in pregnancy. According to Aisling Mannion and Pauline Slade's study, 80% of the samples endorsed at least one item PLEs on the Peters Delusions Inventory, and 76% endorsed at least one item on the Launay–Slade Hallucination Scale—Revised during pregnancy, suggesting that PLEs occur frequently in perinatal individuals without a diagnosis of severe mental illness (10). Another study also showed that psychosis risk is present in pregnancy (9). However, DeVylder and Koyanagi considered that pregnant and peripartum women are not at increased risk for PLEs at the population level (11). Thus, it needs more studies to explore whether psychosis, particularly sub-clinical psychosis (PLEs) became more frequent during the pregnancy phase.

Several pregnancy-related risk factors have been examined in relation to the risk of puerperal psychosis such as birth by cesarean section, primiparity, male gender of the baby, and night-time delivery (12–14). Other psychological factors such as fear of labor, birth trauma, and low social support (15, 16) also have been identified as a risk for puerperal psychosis. According to Hartley's review, anxiety and depression are related to psychotic symptom severity and are also linked with sub-clinical experiences, symptom development, and relapse in non-pregnant population (17). However, the risk factors of PLEs in pregnancy have rarely been studied. It needs more research to explore the risk factors such as anxiety and depression for PLEs in pregnant women.

As noted previously, studies dedicated to PLEs in pregnant women are limited but extremely important. Furthermore, to our knowledge, there has been no research to document PLEs in pregnancy in China. Thus, the aims of this study were (1) to investigate the prevalence and characteristics of PLEs among women without previous psychiatric history in each trimester phase and (2) to explore which variables predict levels of PLEs in pregnant women.

## Methods

### Participants

A multi-center, cross-sectional study was carried out between 20 June and 13 September 2020 in twelve major hospitals, located in the northern (Shandong province and Hebei province), western (Guizhou province and Shanxi province), and central regions of China (Hunan province and Hubei province). These hospitals represent a range of clinical settings in China. Data were collected using the WeChat-based Questionnaire Star application on smartphones. WeChat is a widely used social communication app. Only data from complete questionnaires were analyzed. Pregnant women without previous psychiatric disorders in their first (≤13 week of pregnancy), second (14–27 weeks of pregnancy), or third trimesters (≥28 week of pregnancy) were recruited for the study.

The inclusion criteria for participation in this study were as follows: women in pregnancy, ability to complete the web-based questionnaire on the smartphone by themselves, and voluntary participation. The exclusion criteria were a history of any psychiatric conditions for participants or a family history of psychiatric disorders in order to focus on sub-threshold psychotic experiences and avoid their influences on the PLEs as a previous study (5) or who did not complete the questionnaire independently. This study was approved by the ethics committees of Shenzhen Longhua Maternity and Child Healthcare Hospital and each research ethics committee of the respective hospital.

### Instruments

#### Socio-demographic information

Socio-demographic information and pregnancy history to be collected included maternal age at pregnancy (≤35, 36–39, and ≥40 years), residency status (urban or rural), ethnicity (Han or Minorities), single child status (yes or no), family financial situation (low, middle, and high), highest education attained (junior high and below; high school or technical secondary school; and college degree or above), body mass index (BMI) (<18.5; 18.5–23.9; ≥24 kg/m^2^), pregnancy intentional or not (yes or no), nature conceived or not (yes or no), expectation for baby's gender (yes or no), gravidity (1, 2, ≥3), parity (0, 1, ≥2), number of abortion (0, 1, ≥2), number of fetus (singleton pregnancy or twin pregnancy), factors lead to abortion (without abortion, induced abortion, and spontaneous abortion), planned mode of delivery (planned vaginal delivery, planned cesarean delivery, and depend on the circumstances), smoking status (non-smokers, recent quitters, and smokers), passive smoking exposure in pregnancy (yes or no), and alcohol consumption in pregnancy (yes or no).

#### Community assessment of psychic experiences

The CAPE developed by Stefanis et al. (18) is made up of 42 items that evaluate the Positive (20 items), Negative (14 items), and Depressive (8 items) dimensions of psychotic symptoms in the past 12 months on both a frequency scale (1 = never, 2 = sometimes, 3 = often, 4 = nearly always), and a distress scale (1 = not distressed, 2 = a bit distressed, 3 = quite distressed, 4 = very distressed). The positive subscale of the CAPE represents positive psychotic experiences derived from Peters et al. Delusions Inventory (PDI-21) (19). The positive frequency subscale of CAPE was used to evaluate lifetime PLEs in the past month (20). As some items are redundant, representing similar symptoms (such as “Have you ever heard voices when you were alone?” and “Have you ever heard voices talking to each other when you were alone?”) or are quite common (such as “Have you ever felt as if some people are not what they seem to be?” and “Have you ever felt as if you are destined to be someone very important?”), we selected 8 items guided by previous research (21, 22) to reflect actual delusional and hallucinatory experiences (DEs and HEs). The 8-item Positive Subscale of CAPE potentially could represent the full positive dimensions of CAPE and is a valid and reliable instrument for assessing PLEs in the community, both over a lifetime and in the past month (22, 23). Among these items, six items were related to DEs, and two were related to HEs (**Table 2**). The degree of distress associated with positive symptoms was not addressed in this study.

The Chinese mainland version of CAPE has been translated and validated for some pilot studies and demonstrated good reliability and validity (22, 24).

#### The 7-item generalized anxiety disorder scale (GAD-7)

The GAD-7 developed by Spitzer and colleagues (25) is a self-report instrument designed to assess anxiety and the severity of anxiety symptoms. The item scores range from 0 (not at all) to 3 (nearly every day), resulting in a total score ranging from 0 to 21. Higher scores indicate more severe symptoms of anxiety. The cutoff score for anxiety was set at ≥10, based on the total GAD-7 score (17). The Chinese version of the GAD-7 showed great reliability (Cronbach's alpha = 0.90), and at the optimal cutoff value of 10, a sensitivity of 86.2% and a specificity of 95.5% were calculated (26).

#### Edinburgh postnatal depression scale

The EPDS is a self-report questionnaire designed to screen for depression among women during pregnancy and the postpartum period with good reliability and validity (27). EPDS contains a total of ten items, and each item was divided into four grades (0–3). Possible scores range from 0 to 30, with higher scores indicating greater severity of depressive symptoms. A cutoff score of 10 was considered a positive EPDS screening result as research has validated that a score of 10 or higher has better specificity and sensitivity for major or minor depressive disorder and is useful for screening (28, 29).

### Analyses

Analyses were conducted using IBM SPSS Statistics (Version 22.0; IBM, Inc., Chicago, Illinois). Descriptive statistics were performed for group characteristics. The prevalence was calculated if they had a frequency of “sometimes,” “often,” or “nearly always” on one or more of the eight selected items. The frequency of each item was also counted. Differences in PLEs in each trimester were compared using the chi-square tests. To investigate the predictors of more frequent PLEs and symptoms of anxiety and depression, we first conducted univariate multiple logistic regression analyses to calculate odds ratios (*ORs*) and 95% confidence intervals (95% *CI*). Then, stepwise logistic regression analysis was used for all variables to screen predictors and forward selection was used in the stepwise selection of predictors. A significance level of 0.05 was used for model entry, and a significance level of 0.1 was used for removal. The variance inflation factor was used for the assessment of multicollinearity. The results of multicollinearity diagnosis were showing no-multicollinearity problem for the analysis (tolerance ranged from 0.61 to 0.96 and variance inflation factors ranged from 1.0 to 1.64).

Correlation analysis was conducted through Pearson's correlation coefficient to investigate associations between frequency scores among DEs, HEs, and PLEs and GAD-7 score and EPDS score.

## Results

### Description of the sample

A total of 968 pregnant women agreed to participate in our survey. A total of 9 participants who had a history of psychiatric conditions and 13 who had a family history of psychiatric disorders (4 of them with comorbid previous psychiatric disorders and a family history of psychiatric disorders) were subsequently removed from further analyses, leaving 950 with valid data. These participants were all married without being divorced including 138 women in the first trimester (14.5%), 339 women in the second trimester (35.7%), and 473 women in the third trimester (49.8%). Table 1 shows some of the other social-demographic characteristics. Using the cut-off criteria described previously, the rates of anxiety and depression were 9.2% (9.4% in the first trimester, 8.6% in the second trimester, and 9.5% in the third trimester) and 24.1% (26.8% in the first trimester, 23.0% in the second trimester, and 24.1% in the third trimester), respectively.

**Table 1:** Descriptive statistics of the social-demographic variable.

**Characteristics**	***n* (%)**
Pregnant women (total)	950 (100.0)
First trimester (≤13 w)	138 (14.5)
Second trimester (14–27 w)	339 (35.7)
Third trimester (≥28 w)	473 (49.8)
Maternal age at pregnancy, years	
< 35	834 (87.8)
36–39	96 (10.1)
≥40	20 (2.1)
Residency status	
Urban	711 (74.8)
Rural	239 (25.2)
Ethnicity	
Han	907 (95.5)
Minorities	43 (4.5)
Single child status (Yes)	197 (20.7)
Family financial situation	
Low	147 (15.5)
Middle	366 (38.5)
High	437 (46.0)
Highest education attained	
Junior high and below	169 (17.8)
High school or technical secondary school	178 (18.7)
Junior college or above	603 (63.5)
**BMI (kg/m** ^**2**^ **)**	
First trimester (≤13w)	
< 18.5	20 (14.5)
18.5–23.9	80 (58.0)
≥24	38 (27.5)
Second trimester (14–27 w)	
< 18.5	23 (6.8)
18.5–23.9	188 (55.6)
≥24	127 (37.6)
Third trimester (≥28 w)	
< 18.5	14 (3.0)
18.5–23.9	124 (26.3)
≥24	334 (70.8)
Pregnancy history	
Pregnancy intentional or not (Yes)	606 (63.8)
Nature conceived (Yes)	921 (97.0)
Expectation for baby's gender (Yes)	466 (49.1)
Gravidity	
1	424 (44.6)
2	304 (32.0)
≥3	222 (23.4)
Parity	
0 (primiparity)	483 (50.8)
1	269 (28.3)
≥2	198 (20.8)
Number of abortion	
0	637 (67.1)
1	187 (19.7)
≥2	126 (13.3)
Number of fetuses	
Singleton pregnancy	930 (97.9)
Twin pregnancy	20 (2.1)
Factors leading to abortion	
Without abortion	637 (67.1)
Induced abortion	178 (18.7)
Spontaneous abortion	135 (14.2)
Adverse pregnancy outcome (e.g., stillbirth, dystocia, ectopic pregnancy) (Yes)	79 (8.3)
Disease in pregnancy	210 (22.1)
Planned mode of delivery	
Planned vaginal delivery	505 (53.2)
Planned cesarean delivery	146 (15.4)
Depend on the circumstances	299 (31.5)
Smoking status	
Non-smokers	918 (96.6)
Recent quitters	30 (3.2)
Smokers	2 (0.2)
Passive smoking exposure in pregnancy (Yes)	223 (23.5)
Alcohol consumption in pregnancy (Yes)	8 (0.8)
GAD-7 (≥10)	87 (9.2)
First trimester	13 (9.4)
Second trimester	29 (8.6)
Third trimester	45 (9.5)
EPDS (≥10)	229 (24.1)
First trimester	37 (26.8)
Second trimester	78 (23.0)
Third trimester	114 (24.1)

Gravidity is defined as the number of times that a woman has been pregnant. Parity is defined as the number of times that she has given birth to a fetus with a gestational age of 24 weeks or more, regardless of whether the child was born alive or was stillborn. Disease in pregnancy is defined as previous disease that occurred and persisted in pregnancy before pregnancy and the development of a new disease during pregnancy, such as gestational hypertension. GAD-7, 7-item Generalized Anxiety Disorder scale; EPDS, Edinburgh Postnatal Depression Scale. The cutoff score for anxiety and depression was both set at ≥10, based on the total GAD-7 score and the total EPDS score, respectively.

### Prevalence of PLEs in the sample

Table 2 shows the prevalence of DEs, HEs, and PLEs in this sample. The most common PLE was a delusion of reference (31.5%), followed by a delusion of persecution (17.5%). Approximately a third of the pregnant women in this sample experienced at least one PLE item (PLEs = 37.2%, DEs = 36.6%, and HEs = 7.0%), and more pregnant women experienced PLEs in the first and second trimesters than in the third trimester (66.7 and 66.1%, respectively, vs. 7.8%, *X*^2^ = 347.19, *p* < 0.001). Similarly, more pregnant women experienced DEs (66.0% in the first trimester and 64.9% in the second trimester vs. 7.8%, *X*^2^ = 336.87, *p* < 0.001) and HEs (10.8% in the first trimester and 12.7% in the second trimester, vs. 1.9%, *X*^2^ = 38.61, *p* < 0.001) in first and second trimesters than in third trimester. However, prevalence decreased sharply when the frequency increased to “often” (PLEs = 4.3%, DEs = 4.3%, and HEs = 0.5%). Similarly, more pregnant women experienced frequent PLEs, frequent DEs, and frequent HEs in the first trimester (PLEs = 8.0%, DEs = 8.0%, and HEs = 0.7%) and the second trimester (PLEs = 8.6%, DEs = 8.3%, and HEs = 1.2%) than pregnant women in the third trimester (PLEs = 0.2%, DEs = 0.4%, and HEs = 0.0%). The chi-square test shows that *X*^2^ = 39.51–349.04, *p* < 0.001.

**Table 2:** Frequency of each item in eight items of positive dimensions of the community assessment of psychic experiences (CAPE) and prevalence of different psychotic-like experiences (PLEs) among pregnant women.

	**DEs**							**HEs**			**PLEs**
	**^a^T1**	**^a^T2**	**^a^T3**	**^a^T4**	**^a^T5**	**^a^T6**	**Any**	**^a^T7**	**^a^T8**	**Any**	**Any**
Total											
Prevalence (%)	31.5	17.5	11.5	13.8	13.3	11.1	36.6	6.3	4.3	7.0	37.2
Sometimes (%)	27.5	15.7	10.0	12.3	11.8	9.8	31.0	5.5	3.5	6.1	31.5
Often (%)	3.3	1.3	1.0	1.0	1.1	0.7	4.3	0.5	0.4	0.5	4.3
Nearly always (%)	0.7	0.5	0.5	0.5	0.4	0.6	1.3	0.3	0.4	0.4	1.4
First trimester											
Prevalence (%)	55.1	27.5	19.6	24.0	26.9	20.4	66.0	10.1	6.5	10.8	66.7
Sometimes (%)	48.6	24.6	15.9	21.0	21.0	17.4	55.1	8.7	5.1	9.4	55.8
Often (%)	5.8	2.2	2.2	1.5	4.4	1.5	8.0	0.7	0.7	0.7	8.0
Nearly always (%)	0.7	0.7	1.5	1.5	1.5	1.5	2.9	0.7	0.7	0.7	2.9
Second trimester											
Prevalence (%)	57.0	30.4	19.8	24.2	21.0	20.7	64.9	11.5	7.7	12.7	66.1
Sometimes (%)	49.3	27.1	17.1	21.5	19.2	18.0	54.6	10.0	6.2	10.9	55.5
Often (%)	6.2	2.1	1.8	1.8	1.2	1.5	8.3	0.9	0.9	1.2	8.6
Nearly always (%)	1.5	1.2	0.9	0.9	0.6	1.2	2.0	0.6	0.6	0.6	2.0
Third trimester											
Prevalence (%)	6.3	5.3	3.2	3.4	3.8	1.7	7.8	1.5	1.3	1.9	7.8
Sometimes (%)	5.7	4.9	3.2	3.2	3.8	1.7	7.2	1.3	1.1	1.7	7.2
Often (%)	0.4	0.4	0.0	0.2	0.0	0.0	0.4	0.2	0.0	0.0	0.2
Nearly always (%)	0.2	0.0	0.0	0.0	0.0	0.0	0.2	0.0	0.2	0.2	0.4

CAPE, Community Assessment of Psychic Experiences; PLEs, psychotic-like experiences; PLEs including 6 items DEs and 2 items HEs. DEs, delusional experiences (including T1–6); HEs, hallucinatory experiences (including T7 and T8). a: T1, delusion of reference; T2, delusion of persecution; T3, thought withdrawal; T4, thought insertion; T5, thought broadcasting; T6, a feeling of being controlled; T7, verbal auditory hallucinations; T8, visual hallucinations.

### Factors associated with high frequent DEs, HEs, and PLEs

First, univariate multiple logistic regression was performed to examine the association between socio-demographic factors and pregnant history and PLEs. Rural residency status (*OR* = 1.98), family financial situation (high) (*OR* = 0.34), pregnancy intentional (yes) (*OR* = 2.14), gravidity (2) (*OR* = 0.34), GAD-7 (*OR* = 3.13), and EPDS (*OR* = 4.38) were statistically significantly associated with the frequency of PLEs in this sample. Similarly, rural residency status (*OR* = 1.88), monthly per annual household income (*OR* = 0.37), pregnancy intentional (yes) (*OR* = 2.23), gravidity (2) (*OR* = 0.36), GAD-7 (*OR* = 2.83), and EPDS (*OR* = 4.21) were statistically significantly associated with the frequency of DEs in this sample. Similarly, rural residency status (*OR* = 10.7), GAD-7 *(OR* = 8.27), and EPDS (*OR* = 6.44) were statistically significantly associated with the frequency of HEs in this sample.

Then, stepwise logistic regression analysis was used for all variables to screen predictors. Family economic status as family financial situation was negatively associated with like symptoms in univariate analysis but eliminated by stepwise selection. Gravidity was negatively associated with psychotic-like symptoms in univariate analysis but eliminated by stepwise selection. Rural residency status (*OR* = 1.87), pregnancy intentional or not (no) (*OR* = 2.14) and EPDS ≥10 (*OR* = 3.97) were found to be risk factors of frequent PLEs in this sample, while gravidity (1) (*OR* = 0.28) was found to be protective factors of frequent PLEs. Similarly, pregnancy intentional or not (no) (*OR* = 2.16) and EPDS ≥10 (*OR* = 4.03) were found to be risk factors of frequent DEs, while parity (1) (*OR* = 0.30) were found to be protective factors of frequent DEs in this sample. Similarly, rural residency status (*OR* = 18.6), single child status (8.13), and GAD-7≥10 (*OR* = 8.71) were found to be risk factors for frequent HEs in this sample. The results are presented in Table 3.

**Table 3:** Influential factors of high frequent delusional experiences (DEs), hallucinatory experiences (HEs), and PLEs in pregnant women.

	**OR**	**(95%CI)**	**Pseudo-R2**	**C value**
**Model DEs**			0.1141	0.746
Pregnancy intentional or not				
Yes	1	–		
No	2.16	(1.20, 3.88)^**^		
Parity				
0 (primiparity)	1	–		
1	0.30	(0.13, 0.67)^**^		
≥2	0.72	(0.36, 1.42)		
EPDS				
< 10	1	–		
≥10	4.03	(2.27, 7.16)^***^		
**Model HEs**			0.2555	0.874
Residency status				
Urban	1	–		
Rural	18.6	(3.35, 103.38)^***^		
Single child status				
No	1	–		
Yes	8.13	(1.81, 36.53)^***^		
GAD				
< 10	1	–		
≥10	8.71	(2.12, 35.80)^***^		
**Model PLEs**			0.1299	0.759
Residency status				
Urban	1	–		
Rural	1.87	(1.03, 3.39)^*^		
Pregnancy intentional or not				
Yes	1	–		
No	2.14	(1.19, 3.83)^*^		
Parity				
0 (primiparity)	1	–		
1	0.28	(0.13, 0.63)^**^		
≥2	0.64	(0.32, 1.28)		
EPDS				
< 10	1	–		
≥10	3.97	(2.23, 7.07)^***^		

CI, confidence intervals; OR, odds ratio; DEs, delusional experiences; HEs, hallucinatory experiences; PLEs, psychotic-like experiences; GAD-7, 7-item Generalized Anxiety Disorder scale; EPDS, Edinburgh Postnatal Depression Scale;

* *p* < 0.05,

** *p* < 0.01,

*** *p* < 0.001.

### Anxious and depressive symptoms associated with frequent DEs, HEs, and PLEs

Correlations between frequency scores of DEs, HEs, and PLEs and depressive symptoms were positive and significant (*r* = 0.087, *p* < 0.01; *r* = 0.258, *p* < 0.001 and *r* = 0.081, *p* < 0.05, respectively), and the link between anxious symptoms and HEs was also positive and significant (*r* = 0.126, *p* < 0.001).

## Discussion

To the best of our knowledge, this is the first study to examine the prevalence and correlates of PLEs among pregnant women in China. We found that the prevalence of PLEs was 37.2% among pregnant Chinese women, which suggests that PLEs are particularly common in this population. We also found that 36.6% of participants reported DEs and 7.0% reported HEs, while only 4.3% reported frequent PLEs, 4.3% reported frequent DEs, and 0.5% reported frequent HEs during pregnancy in our study, which suggested that transient PLEs are not pathological. As has been mentioned, the prevalence of PLEs during pregnancy varies greatly among different studies due to the influence of differences in study sites, sample sizes, measurement tools, and cultural background. According to Mannion and Slade's study, 80% of the samples endorsed at least one item of DEs on the Peters Delusions Inventory (PDI), and 76% endorsed at least one item of HEs on the Launay–Slade Hallucination Scale-Revised (LSHS-R) in a recent study on PLEs of the perinatal period (10). Another study conducted in Ghana found that 54.2, 27.3, and 18.5% of participants during pregnancy were at no/low, moderate, and high risk for psychosis, respectively, by using the Prodromal Questionnaire-16 (9). Levey et al. reported that 27% of the 2,059 pregnant Peruvian women scored high on psychosis risk by using the Prodromal Questionnaire-16 (30). However, previous studies addressing this topical issue have mainly focused on the postpartum period, so data on the prevalence of psychosis and PLEs in pregnancy are limited owing to a lack of studies. Only one study concerned with psychosis in Chinese pregnant women showed that 6.83% of 205 pregnant women had psychosis symptoms by using the symptom checklist-90 (31). However, this study major was concerned about psychological symptoms, such as psychosis symptoms, obsessive-compulsive symptoms, depressive symptoms, and so on, but not PLEs.

In our study, pregnant women who experienced PLEs showed a sharp decline in the third trimester compared to the first two trimesters (66.7% in the first trimester and 66.1% in the second trimester vs. 7.8% in the third trimester). Similarly, pregnant women who experienced frequent PLEs also show a sharp decline in the third trimester (8.0% in the first trimester and 8.6% in the second trimester vs. 0.2% in the third trimester). While in Adjorlolo's study, moderate and high risk for psychosis increased from 11.8 and 9.2%, respectively, in the first trimester, to 51.6 and 50.5%, respectively, in the second trimester, but decreased to 36.6 and 40.4%, respectively, in the third trimester (9). According to Fisher's study, the prevalence of common mental disorders in early pregnancy in low-income countries was 22.4% (95% *CI* 18.4–26.4) but dropped to 10.7% in late pregnancy (32). Mannion and Slade reported that endorsement rates of PLEs decreased postnatally compared to pregnancy (10). One possible explanation could be that the PLEs are mostly transient in nature, and the duration of time for PLEs was different across the different time points (20, 23). Previous studies on PLEs of pregnant women are limited. Future studies should compare endorsement rates of PLEs in non-perinatal, pregnant, and postnatal women over equivalent brief time periods.

Variables found to be significantly associated with PLEs in this period were similar to those in the literature on non-puerperal psychosis such as depressive and anxious symptoms. The prevalence of depression during the pregnancy period was 24.1% in this study, compared to 7–15% in high-income countries (33, 34) and 19–25% in low-income and middle-income countries in previous studies (35). Depressive symptomatology predicted delusional-like experiences during pregnancy as well as PLEs in this study. There is increasing data on the link between depression and PLEs in non-pregnant population (36, 37) and pregnant women (9, 10). Cross-sectional studies have shown that adolescents who experienced PLEs increased the risk of having depressive and anxious symptoms than those who never experienced PLEs (36, 38). According to Varghese's study, young adults with depression and anxiety were also more likely to report PLEs symptoms compared with healthy individuals (37). Our study also supports the link between anxious symptoms and HEs. However, the link between anxious symptoms and PLEs has been ignored in previous studies.

Other social demographic variables such as rural household registration showed that it may predict hallucination-like experiences and PLEs similar to previous studies (20, 39). Possible explanations for this finding are lower socioeconomic status, inferior healthcare conditions, lower education levels in rural areas, and more severe social stress than in cities (40). A single-child status was also found to be a risk factor for HEs in this study. A single child is a person with no siblings in his/her family, by birth or adoption. With the purpose to ease the population, the Chinese government launched the “one-child policy” from 1979 to 2013, which allowed each couple to have only one child. The policy was successful in population control, which resulted in hosting a lot of singleton population in China currently. Pregnant women with single child status are 20.7% in this study. A single child with no sibling has a lower tolerance to adversity and increased frustration when presented with challenges (41), which may lead to more psychosis (42). However, a few previous Chinese studies did not find any relationship between single-child status and psychosis (22, 23). More future research needs to focus on this field.

Pregnancy history such as unintentional pregnancy is associated with an increased risk for DEs and PLEs in our study as well as in previous studies (43). According to Fisher's study, unintended pregnancy is a risk factor for determinants of non-psychotic common perinatal mental disorders in low- and lower-middle-income countries (32). Parity (1) has been found to be a protective factor in DEs as well as PLEs. According to Fisher's study, nulli- or primiparity was risk factors for common mental disorders such as depression, anxiety, and somatoform disorder in early pregnancy (44). Jones's review also considered that the consistent finding of risk factors for post-partum psychosis is primiparity although several obstetric factors have been examined in relation to the risk of post-partum psychosis (such as cesarean section, sex of baby, and gestation period) (2). The reason for this is that first pregnancy and the transition to new motherhood might lead to greater psychological stress than subsequent deliveries. However, a study in this field is limited, so further study needs to explore it.

The major strength of this study is the multi-center design with large sample size. Besides, we provided a relatively comprehensive profile of pregnant women's socioeconomic status and pregnancy history to first explore the PLEs in pregnant women of each trimester which were not considered in the previous studies in China. However, there are still some limitations to this study in interpreting the findings. First, the current study is based on self-reported questionnaires which can lead to recall bias. Second, no causal conclusion can be drawn in this study due to the cross-sectional design. Third, a history of any psychiatric conditions for participants or a family history of psychiatric disorders was excluded in order to focus on PLEs as in the previous study; however, these exclusions altered the nature of the sample, given the high prevalence of other psychiatric symptoms and family history of mental health among pregnant women (2, 21), higher prevalence of PLEs in a family history of psychiatric conditions (23), and PLEs is closely related to higher rates of psychiatric disorders, such as bipolar disorder (45). Fourth, the size of some of the 95% *CIs* in model HEs seems too large due to the small sample size for HEs. Future research explores the risk factors for HEs in a larger sample size.

## Conclusion

Episodes of PLEs are common in pregnant Chinese women; however, only a small proportion has persistent PLEs, with the severity of anxious and depressive symptoms increasing as the frequency increased.

## Data availability statement

The raw data supporting the conclusions of this article will be made available by the authors, without undue reservation.

## Ethics statement

The studies involving human participants were reviewed and approved by Shenzhen Longhua Maternity and Child Healthcare Hospital and each Research Ethics Committee of the respective hospital. The patients/participants provided their written informed consent to participate in this study.

## Author contributions

DL, WY, and XiaoqL: study design. SQ, DX, JZ, YZ, and XiaocL: data collection, analysis, and interpretation. DL: drafting of the manuscript. All authors approved the final version for publication.

## Funding

This study was supported by the National Social Science Fund of China (general project for education) (Grant No. BBA170061).

## Conflict of interest

The authors declare that the research was conducted in the absence of any commercial or financial relationships that could be construed as a potential conflict of interest.

## Publisher's note

All claims expressed in this article are solely those of the authors and do not necessarily represent those of their affiliated organizations, or those of the publisher, the editors and the reviewers. Any product that may be evaluated in this article, or claim that may be made by its manufacturer, is not guaranteed or endorsed by the publisher.
